# Dynamics of infection and competition between two strains of *Trypanosoma brucei brucei *in the tsetse fly observed using fluorescent markers

**DOI:** 10.1186/1475-9292-6-4

**Published:** 2007-06-06

**Authors:** Lori Peacock, Vanessa Ferris, Mick Bailey, Wendy Gibson

**Affiliations:** 1 School of Biological Sciences University of Bristol, Bristol BS8 1UG, UK; 2 Department of Clinical Veterinary Science, University of Bristol, Langford, Bristol BS40 7DU, UK

## Abstract

**Background:**

Genetic exchange occurs between *Trypanosoma brucei *strains during the complex developmental cycle in the tsetse vector, probably within the salivary glands. Successful mating will depend on the dynamics of co-infection with multiple strains, particularly if intraspecific competition occurs. We have previously used *T. brucei *expressing green fluorescent protein to study parasite development in the vector, enabling even one trypanosome to be visualized. Here we have used two different trypanosome strains transfected with either green or red fluorescent proteins to study the dynamics of co-infection directly in the tsetse fly.

**Results:**

The majority of infected flies had both trypanosome strains present in the midgut, but the relative proportion of red and green trypanosome strains varied considerably between flies and between different sections of the midgut in individual flies. Colonization of the paired salivary glands revealed greater variability than for midguts, as each gland could be infected with red and/or green trypanosome strains in variable proportions. Salivary glands with a mixed infection appeared to have a higher density of trypanosomes than glands containing a single strain. Comparison of the numbers of red and green trypanosomes in the proventriculus, salivary exudate and glands from individual flies showed no correlation between the composition of the trypanosome population of the proventriculus and foregut and that of the salivary glands. For each compartment examined (midgut, foregut, salivary glands), there was a significantly higher proportion of mixed infections than expected, assuming the null hypothesis that the development of each trypanosome strain is independent.

**Conclusion:**

Both the trypanosome strains used were fully capable of infecting tsetse, but the probabilities of infection with each strain were not independent, there being a significantly higher proportion of mixed infections than expected in each of three compartments examined: midgut, proventriculus and salivary glands. Hence there was no evidence of competition between trypanosome strains, but instead co-infection was frequent. Infection rates in co-infected flies were no different to those found routinely in flies infected with a single strain, ruling out the possibility that one strain enhanced infection with the other. We infer that each fly is either permissive or non-permissive of trypanosome infection with at least 3 sequential checkpoints imposed by the midgut, proventriculus and salivary glands. Salivary glands containing both trypanosome strains appeared to contain more trypanosomes than singly-infected glands, suggesting that lack of competition enhances the likelihood of genetic exchange.

## Background

**Figure 1:**
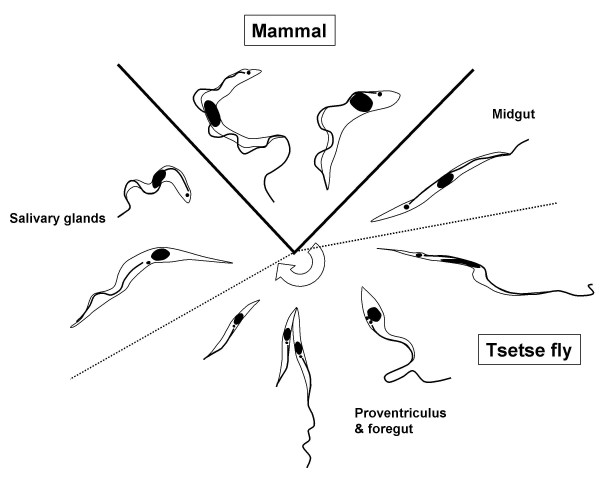
**Developmental cycle of *Trypanosoma brucei***. Diagram illustrating the development cycle of *Trypanosoma brucei *in the mammalian and tsetse fly hosts. The transition between the long trypomastigote found in the proventriculus and the short epimastigote that invades the salivary glands is an asymmetric division [1, 2, 4]. Line drawings traced from fixed Giemsa-stained cells.

*Trypanosoma brucei *undergoes complex cycles of differentiation and multiplication in the tsetse vector. Successful migration from the midgut, where infection is initially established, to the salivary glands via the proventriculus and foregut depends on the ability of trypanosomes to negotiate physical barriers and rapidly adapt to new environmental conditions (Fig 1) [1-4]. As a consequence, trypanosomes experience several population bottlenecks. Mixed infections of two or more trypanosome species occur frequently in wild flies [5,6], but there have been few studies to analyse the occurrence of intraspecific mixed infections either in field [7,8] or laboratory [9,10] flies because of the difficulty of discriminating between trypanosome strains. For genetic exchange to occur, a mixed infection of *T. brucei *sspp. strains in the vector is a prerequisite [11,12], and therefore the dynamics of infection and potential competition between strains must be crucial to the success of this process. Recent advances in the use of fluorescent markers such as Green Fluorescent Protein (GFP) have enabled even a single trypanosome to be identified in the fly [13,14] and allow the dynamics of infection with multiple strains to be easily studied [15-17].

The aim of the present study was to investigate the dynamics of co-infection of tsetse flies with different *T. b. brucei *strains with the specific aim of determining if intraspecific competition occurs among the various developmental stages in the tsetse vector.

## Results

### Midgut infections

Flies were co-infected by feeding with approximately equal numbers of bloodstream forms of two strains of *T. brucei*: J10 red (RFP, red fluorescent protein) and 1738 green (GFP) fluorescent trypanosomes. The total number of flies infected in the whole experimental series was 1734, resulting in a midgut infection rate of 54.5% (945/1734); salivary gland infections were examined in 1663 of these flies that were dissected at least 2 weeks after infection, giving an overall salivary gland infection rate of 3.6% (60/1663). Subgroups of these flies were analysed in various ways, giving rise to the data presented here.

**Figure 2:**
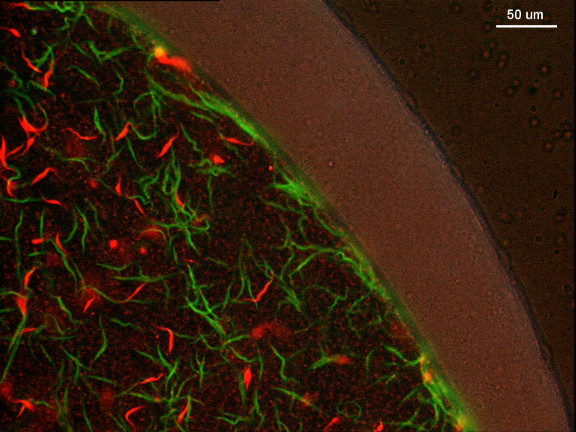
**Mixed trypanosome infection in tsetse midgut**. Fluorescence microscopy image of red and green procyclic trypanosomes in a midgut dissected 3 days after infection. At this stage the trypanosomes are still within the bloodmeal bounded by the peritrophic matrix.

The composition of the trypanosome population was recorded for 411 infected midguts from 771 flies dissected between 3 and 63 days after infection. In our initial analysis, infections were simply categorised into positive or negative for each colour (= strain) of trypanosome. Our null hypothesis was that each trypanosome strain would have an independent chance of establishing an infection; however 396 of the 411 infected midguts (96.4%) were found to contain both red and green trypanosome strains rather than single colour infections (Fig 2; Table 1) and the number of mixed infections was significantly higher than expected (Table 1). The overall midgut infection rate in experimental transmissions of both trypanosome strains was similar to that for single strains (data not shown), ruling out the possibility that one strain enhances infection with the other, and we therefore conclude that co-infection is favoured.

**Table 1:** Summary of midgut infections. Chi squared analysis of numbers of midguts infected with red, green or both trypanosome strains or uninfected (expected numbers in brackets)

	**Red strain**		
**Green strain**	**Infected**	**Uninfected**	Total

**Infected**	396 (211)	7 (192)	403
**Uninfected**	8 (193)	360 (175)	368
Total	404	367	771

× ^2 ^= 711.8, df = 1, *P *≤ 0.001

**Figure 3:**
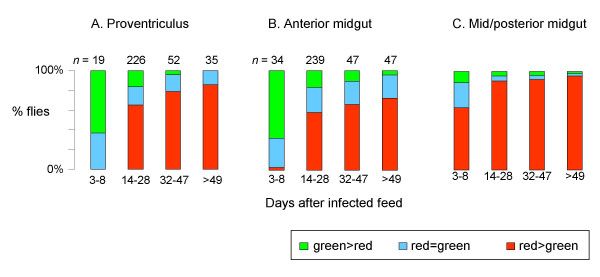
**Analysis of trypanosome infection in midgut sections over time**. Relative proportion of red and green trypanosome strains in different midgut sections over time. In the fly, the proventriculus forms the junction between the foregut and midgut anteriorly; the midgut can then be divided into anterior and mid/posterior sections, as in [29], with the bulk of digestion taking place in the latter section. The Malpighian tubules mark the junction of the posterior midgut and hindgut.

Although most midgut infections were mixed, from the initial analysis it became apparent that the relative number and distribution of the two strains varied considerably both within and between flies. Hence the level of infection with each strain was assessed by categorising trypanosome numbers into low, moderate or high infections, allowing the ratio between red and green trypanosome strains to be determined in different parts of the same midgut and at different times after infection (Fig 3). A 3 × 3 × 4 contingency table was constructed and a 3-dimensional chi-squared analysis was then performed on these ratios (Table 2). From Fig 3 it can be seen that the level of infection with red and green trypanosome strains varied between different sections of the midgut and also varied between flies dissected at different times after infection. The green trypanosome strain was most numerous in the proventriculus and anterior midgut on days 3–8, in contrast to the general pattern of preponderance of the red trypanosome strain, particularly in the mid/posterior midgut. Accordingly, the highest contributions to the overall chi-squared value were from the proventriculus and anterior midgut for the 3–8 day timepoint and the mid/posterior for the 14–28 day timepoint (Table 2). In summary, the distribution of the trypanosome strains within the midgut changed with time and so did the relative parasite load of the two strains.

**Table 2:** Analysis of trypanosome infection in midgut sections over time.

	**Days after infected feed**				
	**3–8**	**14–28**	**32–47**	**≥ 49**	Total

**Proventriculus**					
**Red < green**	19.59*	2.38	2.64	5.16	29.78
**Red = green**	1.24	0.74	0.24	0.39	2.60
**Red > green**	19.30*	0.32	2.52	0.20	22.34
Total	40.13	3.44	5.40	5.75	54.72
					
**Anterior midgut**					
**Red < green**	90.89*	2.14	0.39	2.40	95.82
**Red = green**	4.72	9.20	0.77	1.88	16.56
**Red > green**	19.38*	5.71	0.55	0.39	26.03
Total	114.98	17.05	1.70	4.67	138.41
					
**Mid/posterior midgut**					
**Red < green**	0.01	13.26	3.14	3.81	20.22
**Red = green**	3.11	21.23*	4.84	5.36	34.53
**Red > green**	0.04	12.94	1.82	4.60	19.40
Total	3.15	47.43	9.81	13.77	74.16

Each 3 × 4 table contains the chi-squared values obtained for individual cells for each part of the midgut using the data shown in Fig 3. * indicates highest contributions to the overall chi-squared value for each midgut section

### Proventriculus infections

**Figure 4:**
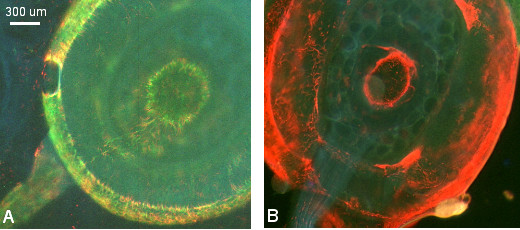
**Proventriculus infected with trypanosomes**. Fluorescence microscopy images of trypanosome-infected proventriculi. A. proventriculus containing both red and green trypanosomes. B. proventriculus containing predominantly red trypanosomes.

From the proventriculus (Fig 4), trypanosomes migrate anteriorly to reach the salivary glands. Table 3 compares the composition of the trypanosome population in the proventriculus and salivary glands in a total of 331 flies. Although the red trypanosome strain appeared to preponderate in the proventriculus (Table 3), there was no significant relationship between the ratio of red and green trypanosomes in the proventriculus and the colour (= strain) of trypanosomes in the infected glands (× ^2 ^= 6.4, df = 4, *P *= 0.172). Neither was there a significant association between the ratio of red and green trypanosomes in the proventriculus and whether or not the fly had a salivary gland infection (× ^2 ^= 3.6, df = 2, *P *= 0.166). Thus, despite numerical superiority of the red trypanosome in the midgut and proventriculus, it appeared to have no advantage in terms of overall ability to reach the salivary glands.

**Table 3:** Comparison of infections in proventriculus and salivary glands in individual flies

Trypanosome strain(s) in salivary glands	Ratio of red and green trypanosome strains in proventriculus		
	
	Red > green	Red = green	Red < green
Green only	6	1	4
Red & green	11	1	1
Red only	7	0	0
**Total no. of flies with infected salivary glands**	**24**	**2**	**5**
**Total no. of flies with uninfected salivary glands**	**193**	**61**	**46**
Total	217	63	51

### Foregut infections

**Figure 5:**
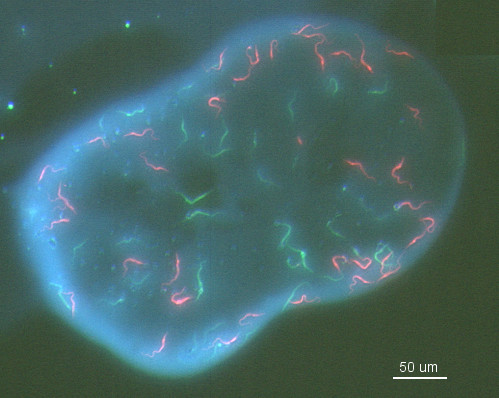
**Trypanosomes in salivary exudates**. Fluorescence microscopy image of red and green trypanosomes extruded by an individual fly during probing onto a microscope slide.

Foregut developmental stages were assayed by examination of salivary exudates from individually caged flies from day 8 to 28 after infection, allowing the colour (= strain) of trypanosomes to be recorded (Fig 5). The salivary exudate is produced by hungry flies as they probe and is a mixture of saliva and fluid regurgitated from the foregut [1]. At the end of the experiment, all flies were dissected and 202 had a midgut infection; 58 of the 202 midgut-infected flies (29.1%) had extruded trypanosomes in the saliva, but this had culminated in a salivary gland infection in only 15 of the 58 flies (25.9%). In Table 4 the composition of the trypanosome population in the salivary exudate is compared with that in the salivary glands for each individual fly; the numbers are too small for statistical analysis, but the composition of the two populations is broadly in agreement.

**Table 4:** Trypanosomes in foregut (salivary exudate) versus salivary glands. Breakdown of infections in individual flies.

Trypanosome strain(s) in paired salivary glands of individual flies	Trypanosome strain(s) in salivary exudate (no. of flies)			Total
		
	Red & green	Green only	Red only	
Both mixed	1	0	0	1
1 mixed,1 green	3	1	0	4
1 mixed,1 red	1	0	0	1
Both green	0	2	0	2
Both red	0	0	0	0
1 red, 1 green	1	0	1	2
1 mixed, 1 uninfected	0	0	0	0
1 red, 1 uninfected	1	0	2	3
1 green, 1 uninfected	2	0	0	2
**Total no. of flies with infected salivary gland**	**9**	**3**	**3**	**15**
**Total no. of flies with uninfected salivary gland**	**25**	**10**	**8**	**43**
Total	34	13	11	58

The salivary exudate from 34 of 58 flies contained a mixture of the red and green strains (Table 4). Again, we assumed a null hypothesis that each trypanosome strain would have an independent chance of being found in the salivary exudate; however the number of flies extruding both red and green trypanosome strains was significantly higher than expected, indicating that the probabilities of the two strains successfully infecting the foregut were not independent (Table 5). There was no significant association between the trypanosome composition of the exudate and whether or not flies developed a salivary gland infection (× ^2 ^= 0.070, df = 2, *P *= 0.965), so it was not the case that one strain was more successful than the other in establishing a salivary gland infection.

**Table 5:** Trypanosomes in foregut (salivary exudate) versus salivary glands. Chi squared analysis of numbers of flies with salivary exudate containing red, green or both trypanosome strains or none (expected numbers in brackets). Data from Table 4

**Green strain**	**Red strain**		
	
	**Infected**	**Uninfected**	Total
**Infected**	34 (10)	13 (37)	47
**Uninfected**	11 (35)	144 (120)	155
Total	45	157	202

× ^2 ^= 88.7, df = 1, *P *≤ 0.001

### Invasion of salivary glands

**Figure 6:**
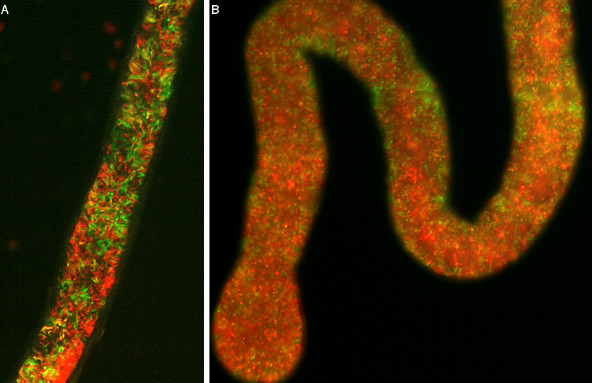
**Patchy distribution of trypanosomes in salivary glands**. Fluorescence microscopy images of salivary glands dissected at 4 weeks post infected feed, showing patchiness of the distribution of red and green trypanosomes along the gland.

Each tsetse fly has two salivary glands. Table 6 shows the composition of the trypanosome population in the paired salivary glands of 60 individual flies. Less than half of these flies had matching infections in both salivary glands of the pair (43.3%; 26/60), and four flies had red trypanosomes in one gland and green in the other. Frequently, only one gland of the pair was infected (36.7%; 22/60). These observations suggest that each gland is invaded and colonized separately. It appears that relatively few trypanosomes serve as the founder population for each gland, as some gland infections contained only small numbers of trypanosomes. For example, seven flies had 2–5 trypanosomes per gland when dissected 2–4 weeks after infection. This may result from small numbers of invading trypanosomes or poor success in colonization of the gland. Within the gland trypanosomes appear to attach to the salivary gland epithelium at random positions and then multiply, as red and green trypanosomes often showed a patchy distribution along the length of the gland (Fig 6). Infection of both glands or with both trypanosome strains did not appear to depend on the duration of infection (Table 6). For example, flies with a mixed infection of both glands were found from weeks two to nine, while in some flies one gland was still uninfected on dissection at seven weeks.

**Table 6:** Summary of salivary gland infections. Breakdown of infections in individual flies

**Trypanosome strain(s) in paired salivary glands**	**Total no. of flies**	**No. of flies with infected salivary glands (week of dissection)**							
		
		**2**	**3**	**4**	**5**	**6**	**7**	**8**	**9**
Both mixed	13	6	2	2	1		1		1
1 mixed, 1 green	5	1	1	3					
1 mixed, 1 red	3			1			2		
1 mixed, 1 uninfected	1			1					
1 green, 1 red	4	1	1	2					
Both green	11	1	2	1	3	1	1	1	1
Both red	2			1			1		
1 green, 1 uninfected	11		1	3	6		1		
1 red, 1 uninfected	10		6	3			1		
**Total**	**60**	**9**	**13**	**17**	**10**	**1**	**7**	**1**	**2**

From these observations it appears that invasion and colonisation of each gland of the pair should be considered as an independent event and Table 7 shows the infections broken down in this way according to strain. Again we assumed a null hypothesis that each strain had an independent chance of reaching and establishing infection in a gland; however the number of mixed gland infections was significantly higher than expected (Table 7), indicating that the probabilities of gland infection with the two strains were not independent.

**Table 7:** Summary of salivary gland infections. Chi squared analysis of numbers of salivary glands containing red, green or both trypanosome strains or none (expected numbers in brackets). Data from Table 6

**Green strain**	**Red strain**		
	
	**Infected**	**Uninfected**	Total
**Infected**	35 (2)	42 (75)	77
**Uninfected**	21 (54)	1714 (1681)	1735
Total	56	1756	1812

× ^2 ^= 481.9, df = 1, *P *≤ 0.001

### Pixel-counting of trypanosomes in salivary glands

**Figure 7:**
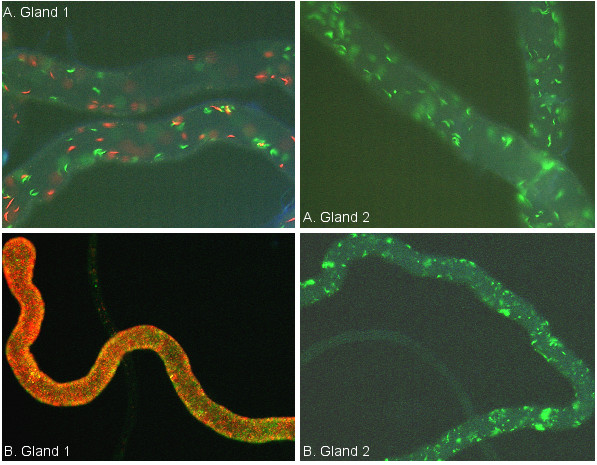
**Comparison of paired salivary glands from a single fly**. Paired salivary glands from individual flies dissected at (A) 2 weeks, and (B) 4 weeks post infected feed. Gland 1 of each pair has a mixed infection and gland 2 a single infection. At the early stage of establishment (A), both glands have about the same density of trypanosomes, while at the later stage (B), trypanosomes in the gland with the mixed infection appear to be more dense than in the gland with the single infection.

In infections of the same duration, glands with a mixed infection appeared to have a substantially heavier parasite load than glands infected with only a single trypanosome strain; this was particularly obvious for non-matching glands, e.g. Fig 7. This impression was confirmed by analysis of the digital images using automated pixel counting [18]. The total number of pixels in a section of gland was determined, together with numbers of red, green and yellow pixels; the proportion of pixels of a particular colour was then used to deduce the area of gland occupied by trypanosomes of that colour. Since co-localization analysis showed that the observed number of yellow pixels was not significantly greater than expected, yellow pixels were assumed to derive from overlapping green and red trypanosomes rather than yellow hybrids and were therefore added equally to red and green pixel counts, ie. one yellow pixel = one green and one red pixel.

**Figure 8:**
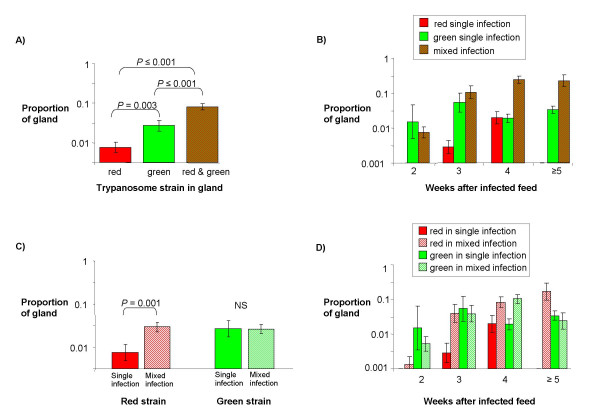
**Analysis of density of trypanosomes in salivary glands**. Histograms comparing the proportion of the salivary gland occupied by trypanosomes in infections with single or both red and green strains. A total of 63 glands from 41 individual flies were analysed, comprising 27 glands with only green trypanosomes, 11 glands with only red trypanosomes and 25 glands with a mixed infection. Bars show geometric means and geometric standard errors. (A) Proportion of the gland occupied by trypanosomes in single infections compared to mixed infections. There were significantly more trypanosomes present in glands containing the green strain compared to the red strain, and significantly more trypanosomes present in glands with mixed compared to single infections. (B) Comparison of salivary glands dissected at different timepoints shows that trypanosomes became more dense in glands with a mixed infection compared to a single strain infection after 3 weeks duration of infection. (C) Proportion of the gland occupied by the same trypanosome strain in a single compared to a mixed infection. There were significantly more trypanosomes of the red strain present in glands containing the green trypanosome strain as well; the converse was not true for the green strain. (D) Comparison of salivary glands dissected at different timepoints shows that this effect became pronounced for the red strain from 2 weeks duration of infection.

Images of infected glands dissected between 2 and 9 weeks after infection were used for pixel counting. A total of 63 glands from 41 individual flies were analysed, comprising 27 glands with only green trypanosomes, 11 glands with only red trypanosomes and 25 glands with a mixed infection. Overall, glands with a mixed infection had a highly significant larger average total area of trypanosomes than glands infected with a single strain (*P *< 0.001, Tukey *post-hoc*) (Fig. 8A). The total area of trypanosomes increased significantly with duration of infection (*P *< 0.001, ANOVA), with a significant correlation between the duration of infection and whether the infection was single or mixed (*P *= 0.004, ANOVA). The area of trypanosomes in glands with a mixed infection increased rapidly from three weeks, whereas the total area in glands with a single strain remained relatively low (Fig 8B). Glands with a mixed infection had a significantly higher area of red trypanosomes than did glands containing the red strain only (*P *= 0.001, ANOVA) (Figs 8C and 8D). Although, a corresponding difference was not observed for the green trypanosome strain (*P *= 0.967, ANOVA) (Fig 8C), there was a significant interaction between the duration of infection and whether the infection was single or mixed (*P *= 0.030, ANOVA) (Fig 8D), especially evident at four weeks after the infected feed.

## Discussion

The use of fluorescent markers has enabled us to study the dynamics of co-infection of tsetse flies with two different strains of *T. b. brucei*. We found no evidence that one trypanosome strain out competed the other strain in terms of the relative success in establishing infection in different parts of the tsetse alimentary tract. For *T. brucei*, competition between strains would decrease the chances of co-transmission and hence genetic exchange, which takes place during tsetse transmission [11,12]. Therefore the apparent lack of competition may be advantageous in enhancing opportunities for genetic exchange, and indeed these two strains produced hybrids when they occurred together in a salivary gland [19]. On the other hand, the fitness of a particular trypanosome strain can be judged on the ability to be transmitted [20], and viewed from this perspective, the two trypanosomes were equally fit and thus neither strain had a competitive advantage.

The results do however provide evidence of partitioning of strains within the midgut, possibly indicating an interaction between the two strains. The observed differences in relative distribution of the two trypanosome strains over time (especially during early establishment) was unexpected and suggests either that different strains favour different parts of the midgut or that the two strains compete for space/resources. While the green strain was more prevalent in the anterior midgut and proventriculus, the red strain was numerically dominant, especially in the mid/posterior section. Neither situation appeared to give a transmission advantage, since the foregut and salivary gland infection rates for both strains were about the same. However, it is possible that the difference in numbers and relative distribution reflect different strategies allowing each strain to 'hold its own' within the insect. Maintaining infection at a high level seems guaranteed to enhance the chance of onward migration (red strain), but early positioning in the proventriculus and anterior midgut should also favour passage through the foregut, independent of overall trypanosome numbers (green strain). It is not clear from these experiments whether the two strains have intrinsic differences or adopted different strategies because of co-infection. Partitioning is known to occur among different trypanosome species in the tsetse fly as different species have different niches for maturation. For example, whereas *T. brucei *moves anteriorly from the midgut to the foregut and salivary glands, *T. grayi*, a tsetse-transmitted parasite of crocodiles, migrates posteriorly and completes its development in the hindgut. Partitioning of *T. brucei *strains might enable strains to optimise efficient use of resources in the host and thus maximise the chances of co-transmission and genetic exchange.

Rather than competition, our results suggest cooperation as there were significantly higher numbers of mixed midgut, foregut and salivary gland infections than expected, showing that the probabilities of infection with the two strains were not independent at any stage of development. For example, while trypanosomes established a midgut infection in just over half the flies, almost all midgut infections were mixed. Similarly, less than a third of infected flies extruded foregut developmental stages, but nearly two thirds of these flies showed a mixture of trypanosomes in the extruded sample. Finally, while less than 7% of midgut infections gave rise to a salivary gland infection, both trypanosome strains were present in almost half of these salivary gland infections. Moreover, analysis of the relative proportion of the two strains in the salivary glands showed that significantly greater numbers of trypanosomes were present in glands with a mixed rather than single strain infection, irrespective of the duration of infection.

Since the two trypanosome strains were of equal fitness in terms of transmissibility with similar numbers of single colour infections in the midgut, foregut and salivary glands, it is unlikely that one trypanosome strain enhanced infection with the other. Our observations are more consistent with the hypothesis that individual flies are either permissive (susceptible) or non-permissive (resistant) to infection, and that permissiveness operates at a series of gates that are encountered sequentially by invading trypanosomes as they progress in their developmental cycle through the fly. Firstly, the establishment of a midgut infection, with subsequent invasion of the ectoperitrophic space and proventriculus, relies on trypanosomes surviving the initial immune response of the fly [3,21-23]; in our experiment, about half of the infections foundered at this stage. Secondly, proventricular trypanosomes need to differentiate into migratory forms and invade the foregut by retraversing the peritrophic matrix; here, over two thirds of established midgut infections failed to progress beyond the proventriculus. The obstacles to be overcome by the trypanosome at this stage have been little studied as yet, but may include specific immune responses in the proventriculus [22,24] or foregut, or possibly a physical barrier in terms of the relative difficulty of crossing the peritrophic matrix in some flies or failure to produce migratory developmental forms [1-4]. Thirdly, in many flies with demonstrable foregut infections, the migratory trypanosomes either fail to reach the salivary glands, or, once there, fail to establish an infection, perhaps again because of a specific immune response in the salivary glands. Here, nearly three quarters of foregut infections did not result in an established salivary gland infection.

The suggestion that a fly is either permissive or non-permissive to *T. brucei *infection concurs with results for interspecies studies using *T. b. brucei *and *T. congolense *in tsetse [9,10,25]. If translated to the natural transmission cycle, this has interesting implications in explaining why many flies have mixed trypanosome infections despite low infection rates overall [5,6]. If permissive flies amass infections with multiple trypanosome species, then as suggested previously [26], only a small fraction of the tsetse population may actually pose a risk in terms of transmission of trypanosomiasis.

## Conclusion

We found no evidence of competition between trypanosome strains; on the contrary, there were significantly more mixed infections than expected in the midgut, foregut and salivary glands. This suggests that both trypanosome strains used were fully capable of infecting tsetse, but the flies were either permissive or non-permissive for infection. Barriers to infection were evident at 3 sequential checkpoints: midgut, proventriculus and salivary glands.

Salivary glands containing both trypanosome strains appeared to contain more trypanosomes than singly-infected glands. We speculate that the lack of competition between trypanosome strains serves to enhance the likelihood of genetic exchange.

## Methods

### Tsetse flies

Experimental tsetse flies were from the Bristol laboratory colony of *Glossina morsitans morsitans *originally from Zimbabwe. Flies were kept at 25°C and 70% relative humidity, and fed on sterile defibrinated horse blood via a silicone membrane; bloodmeals for infected flies were supplemented with 2.5% w/v bovine serum albumen (Sigma A4503) [27] and 1 mM dATP [28]. Male flies were used for experiments, being given the infective bloodmeal for their first feed 24–48 hours post-eclosion. The infective bloodmeal contained approximately equal numbers of bloodstream form trypanosomes of each strain (approximately 8 × 10^6 ^trypanosomes ml^-1^) in sterile horse blood supplemented with 60 mM N-acetylglucosamine [29]. For examination of trypanosomes extruded in saliva samples, flies were caged individually; for other experiments, flies were caged in groups of 25–30.

### Trypanosomes

The trypanosome clones used were 1738 (*T. b. brucei *MCRO/KE/72/1738; [30]) transfected with a gene for EGFP and J10 (*T. b. brucei *MCRO/ZM/72/J10 CLONE 1; [31]) transfected with a gene for modified RFP [32]. These two clones are referred to as the green and red trypanosome, respectively. Details of constructs and transfection are given in [13,19].

### Midgut dissection

Flies were dissected 3 to 63 days after infection. Whole tsetse alimentary tracts, from the proventriculus to the rectum, were dissected in a drop of phosphate buffered saline (PBS) and arranged lengthways for assessment of fluorescence. The presence of red and/or green trypanosomes was noted, then each gut section was scored for the relative amount of red and green trypanosomes on a 3-point scale: low (none or negligible), moderate (some or all of section covered, individual trypanosomes discernible), high (section densely filled, individual trypanosomes not discernible). The reliability of this subjective scoring system was assessed by carrying out trypanosome counts on a sample of flies as described by [29].

### Saliva samples

Saliva samples were obtained from individually caged flies essentially as described by [1]. Flies were starved for approximately 48 hours before being allowed to probe onto an alcohol-cleaned microscope slide on a heating plate held at approximately 37°C; flies were removed once they had probed, or after a maximum of about 30 minutes, and then given a blood feed. The cycle of starvation, probing and feeding was repeated to provide saliva samples on alternate days from 7 to 28 days. Saliva samples dried immediately on contact with the microscope slide and slides were stored in the dark at ambient temperature for up to two days before examination. Saliva samples were checked for presence of trypanosomes under brightfield (x100 magnification) and positive samples were subsequently viewed by fluorescence imaging to record the colour of the parasites using a DMRB microscope (Leica) equipped with a Colour Coolview camera (Photonic Science) and ImagePro Plus software (Media Cybernetics).

### Salivary gland infections

Whole salivary glands from flies infected for 13+ days were dissected into a drop of PBS and viewed as wet mounts under bright field illumination (x100 magnification) to search for trypanosomes. Positive glands were then viewed by fluorescence microscopy to determine the colour of trypanosomes present inside each gland. For estimation of the numbers of red and green trypanosomes present and their relative abundance, a computer assisted pixel-based analysis was used [18]. The digital images were analysed using Image J software [33]. A macro was first run to compensate for the bleeding of red into the green channel (determined to be on average 0.3 from an analysis of a gland infected with the green trypanosome only). The image was then sharpened and thresholds of green and red determined to assess the intensity of background colours. Using the original image, the total number of pixels in the area of the gland under analysis was counted automatically, together with the numbers of green, red and yellow pixels; the yellow pixels represent either co-localizing red and green cells or hybrid cells containing both fluorochromes. Co-localization analysis [18] was carried out to determine whether the observed number of yellow pixels differed significantly from the value expected for co-localizing cells.

### Statistical analyses

The chi-squared test was used for comparison of relative presence of red and green trypanosomes, the null hypothesis being that each trypanosome strain would have an independent chance of establishing an infection. ANOVA was used for comparison of proportion of gland with red/green trypanosomes, and proportion of total red and green trypanosomes in mixed or single colour glands. Trypanosome proportions were log transformed prior to analysis to normalise variances. *Post-hoc *tests (Tukey) were performed on significant effects. All ANOVA data were processed using the statistical package SPSS version 12.0 and chi-squared data processed using Excel spreadsheets or [34].

## Competing interests

The author(s) declare that they have no competing interests.

## Authors' contributions

LP and VF carried out the tsetse transmission experiments and imaging; WG prepared the trypanosomes; LP and MB carried out statistical analysis; WG and LP designed the study and drafted the manuscript; MB advised on image analysis and contributed to writing the paper. All authors read and approved the final manuscript.
